# Diagnose und Differenzierung von kutanen Zysten mit optischer Kohärenztomographie: eine Fallserie

**DOI:** 10.1007/s00105-023-05274-8

**Published:** 2023-12-18

**Authors:** Sarah Hobelsberger, Frank Friedrich Gellrich, Julian Steininger, Stefan Beissert, Jörg Laske

**Affiliations:** Department of Dermatology, Faculty of Medicine, Universitätsklinikum Carl Gustav Carus, Technische Universität Dresden, Fetscherstr. 74, 01307 Dresden, Deutschland

**Keywords:** Optische Kohärenztomographie, Trichilemmalzyste, Hidrozystom, Mukoide Pseudozyste, Epidermalzyste, Optical coherence tomography, Trichilemmal cyst, Hidrocystoma, Mucoid pseudocyst, Epidermal cyst

## Abstract

Kutane zystische Läsionen (*n* = 35) wurden mit optischer Kohärenztomographie untersucht. Zysten waren sichtbar als hyporeflektive rundliche Raumforderung mit klarer Abgrenzung unter teils verdünnter Epidermis. Epidermalzysten, trichilemmale Zysten und Hidrozystome hatten einen linearen Rand, der das Zystenepithel darstellt, während mukoide Pseudozysten keinen linearen Rand aufwiesen. Trichilemmal- und Epidermoidzysten wiesen zudem einen hyperreflektiven Inhalt auf, welcher Keratin entspricht. Durch die Visualisierung des Randsaums und des Inhalts der Zyste war es möglich, zwischen verschiedenen Entitäten von Zysten zu differenzieren.

## Abstract (en)

Cutaneous cystic lesions (*n* = 35) were examined with optical coherence tomography. Cysts were visible as a hyporeflective roundish area with a clear margin; in some cases, the epidermis was thinned. Epidermal cysts, trichilemmal cysts, and hidrocystomas had a linear margin representing the epithelium of the cyst, whereas mucoid pseudocysts showed no linear margin. Trichilemmal and epidermal cysts presented with hyperreflective content that corresponds to keratin. By visualizing the margin and the content of the cyst, it was possible to differentiate between different types of cysts.

Die optische Kohärenztomographie (OCT) ist ein nichtinvasives bildgebendes Verfahren, das einen Diodenlaser zur Visualisierung der Haut verwendet [1, 2]. OCT erzeugt horizontale und vertikale Bilder der Haut und wird für die Frühdiagnose von Basalzellkarzinomen, Plattenepithelkarzinomen und deren Vorstufen eingesetzt [1–3]. Da in der OCT auch die Dermis bis zu einer Tiefe von etwa 1,5 mm dargestellt werden kann, haben wir das Verfahren für die Untersuchung von kutanen Zysten eingesetzt [1, 2].

## Methodik

Im Universitätsklinikum Carl Gustav Carus Dresden, Deutschland, wurden 31 Patienten mit 35 zystischen Läsionen mit OCT (VivoSight® [Michelson Diagnostics, Kent, UK]) untersucht. Die Studie wurde in Übereinstimmung mit der Deklaration von Helsinki und mit Genehmigung der örtlichen Ethikkommission (BO-EK-152042022) durchgeführt.

## Ergebnisse

Untersucht wurden 17 Hidrozystome, 9 mukoide Pseudozysten, 2 trichilemmale Zysten und 7 Epidermalzysten. Histopathologisch diagnostiziert wurden 5 Hidrozystome, 1 mukoide Zyste, 2 trichilemmale Zysten und 3 Epidermalzysten. Die Läsionen befanden sich am Augenlid (18), an der Nase (2), an der Glabella (1), am Rumpf (1), am Oberkopf (3) und an den Extremitäten (10).

**Figure 1:**
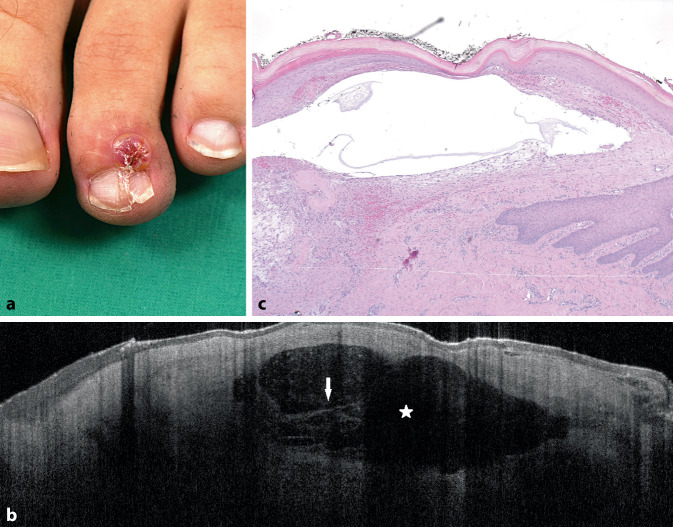

Wir berichten hier über die wichtigsten Merkmale der Zysten in der OCT, die wir in 35 Fällen beobachtet haben (Abb. 1 und 2):

**Figure 2:**
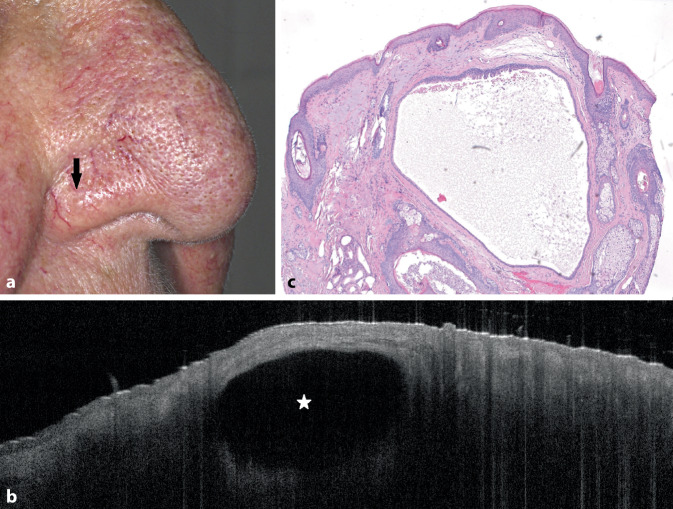

Zystische Läsionen waren sichtbar als schwarze runde oder ovale Raumforderung mit klarer Abgrenzung (Abb. 1 und 2, Tab. 1). In den meisten Fällen war die Epidermis unverändert, in manchen Fällen war die Epidermis über dem Zentrum der Zyste verdünnt.

**Table 1:** 

OCT	Epidermalzyste *n* = 7	Trichilemmalzyste *n* = 2	Hidrozystom *n* = 17	Mukoide Pseudozyste *n* = 9
Runde oder ovale Raumforderung mit klarer Abgrenzung	X	X	X	X
Schwarzer Inhalt der Raumforderung	–	–	X	X
Homogener hyperreflektierender Inhalt der Raumforderung	X	X	–	–
Unterteilung der Raumforderung mit Septen in Kompartimente	X	X	–	X
Linearer Rand	X	X	X	–

Einige Zysten hatten einen zentralen homogenen hyperreflektierenden Inhalt und/oder waren mit hyperreflektierenden Linien als Septen in Kompartimente unterteilt (Abb. 1, Tab. 1). Bei Patienten mit multiplen Hidrozystomen waren mehrere schwarze rundliche Areale sichtbar. Epidermalzysten, trichilemmale Zysten und Hidrozystome hatten einen linearen Rand, der dem Epithel entspricht, während mukoide Pseudozysten keinen linearen Rand aufwiesen (Tab. 1). Trichilemmal- und Epidermalzysten wiesen einen linearen Rand und einen hyperreflektiven Inhalt der Raumforderung auf, der korrespondierend zum histologischen Befund Keratin entspricht (Tab. 1).

## Diskussion

Zystische Läsionen der Haut sind in den meisten Fällen gutartige Läsionen, die klinisch diagnostiziert werden können [4, 5]. Dennoch stellen sie manchmal eine diagnostische Herausforderung dar (Abb. 2). So wurden mehrere der untersuchten Läsionen von niedergelassenen Dermatologen mit Verdacht auf Basalzellkarzinom in unsere Klinik überwiesen (Abb. 2). In der klinischen Routine wird die Sonographie für die Diagnostik von kutanen und subkutanen Zysten eingesetzt [4–6]. Vor allem kleine Zysten wie Hidrozystome an speziellen Lokalisationen wie dem Augenlid lassen sich mit OCT besser untersuchen. Durch die Visualisierung des Randsaums und des Inhalts der Zyste war es möglich, zwischen verschiedenen Entitäten zu unterscheiden. Willard et al. verwendeten in vivo konfokale Lasermikroskopie (RCM) zur Unterscheidung eines Hidrozystoms von einem Basalzellkarzinom [5]. Eine Limitation der OCT ist die geringe Auflösung [1, 2]. Verfahren wie RCM und Line-field-konfokale OCT (LC-OCT) generieren Bilder mit einer zellulären Auflösung und könnten durch Darstellung der Epithelzellen zu einer besseren Differenzierung zwischen verschiedenen Entitäten von Zysten führen [5, 7–9]. Allerdings ist bei diesen Verfahren die Eindringtiefe deutlich geringer (RCM: bis zu 250 μm, LC-OCT: bis zu 500 μm) als bei der OCT (bis zu 1,5 mm), sodass bei größeren Zysten mit diesen Verfahren nur der oberste Anteil der Zyste dargestellt werden kann oder tiefer liegende Zysten übersehen werden könnten [1, 2, 7–9]. In der vorliegenden Studie konnten bei klinisch unklaren Läsionen drei Exzisionen vermieden werden.

## Fazit für die Praxis

Kutane zystische Läsionen sind mit OCT gut darstellbar. Die Darstellung des Zysteninhalts und des vorhandenen Zystenepithels erlaubte in einigen Fällen, zwischen verschiedenen Zystenentitäten zu unterscheiden. OCT könnte unterstützend zu Methoden wie Ultraschall ein hilfreiches Tool in der Diagnostik von kutanen Zysten sein und bei der Unterscheidung zwischen Zysten und malignen Tumoren helfen.
